# An Inquiry into the Degree of Certainty in Medicine; and into the Nature and Extent of Its Powers over Disease

**Published:** 1848-11

**Authors:** 


*An Inquiry into the degree of Certainty in Medicine; and into the Nature and Extent of its powers over Disease.* By Elisha Bartlett, M. D., Professor of the Practice of Medicine in Transylvania University; Member of the American Academy of Arts and Sciences, etc. etc, pp. 84. Lea & Blanchard, Philadelphia: 1848.

This is a curious production, the like of which we have seldom seen from the pen of any one who had passed the age of a sophomore. What makes it the more remarkable, is the circumstance, that the writer is a gentleman of education and experience, and the author of works which have given him a wide reputation. We shall attempt neither analysis nor criticism of the essay; but that our readers may see what a learned and grave Professor *can* write, when he is in the mood, we quote a few passages, which may be regarded as fair specimens of the whole—so like the rest, indeed, that the difficulty is to know what to extract as the most characteristic.

In the preface we have the following apology, or attempt at apology, for the style in which the work is written. It reminds one of the sailor's apology for swearing—" I know it's bad to swear, your Reverence, but it's *damn'd* hard to help it."

" Some of my readers, especially the more staid and older ones —my esteemed and venerable seniors and cotemporaries—may think, perhaps, that I have now and then suffered myself to be seduced into dangerous proximity to the boundary line which separates good taste and simplicity from their opposite qualities. If I have done so, it has been from yielding to an impulse, excited and kindled by the task in which I have been engaged, and which I found it difficult to resist. If the drapery of our philosophic muse wears a somewhat warmer and livelier hue than the sober colouring which more appropriately belongs to it, it has been caught from the sunny fields through which her pathway has lain. If the axle of our car has now and then waxed fervid, and if an occasional gleam of light has flashed from its flying wheels, let us at least plead, in extenuation of the weakness, that it is not from the careless rein, or any perilous speed with which our coursers have been driven, but from the glowing and radiant atmosphere through which we have been carried along.

" There may, possibly, be others, who will say that my advocacy of the claims of medicine is more zealous and earnest than is becoming in one of its practitioners and teachers. I can only answer, that I do not think so. I speak for the art and the science, not for myself. This art and science have been violently, and I think, blindly and unjustly assailed, by parties who understand neither their own strength and position, nor ours. I have endeavoured to make fair and manly stand against them, and I have done nothing more. When crowds of epauletted and bedizened coxcombs, who have never smelt gunpowder, make the air clamorous with their noisy boastings, the war-worn and scar-covered veteran may, at least, point to the trophies that he has brought from a hundred battle-fields."

In the course of his argument on "the degree of certainty in medicine," the author introduces pneumonia, and the results of various modes of its treatment, when we meet with the following logical conclusions :

"Let us endeavour to see what is the precise character of the evidence contained in the foregoing statements, that we can control and cure pneumonia. Let us analyze, as far as we can, this evidence, and reduce it to its elements, in order to determine its nature, and to fix its degree of positiveness.

In the first place, it is entirely certain that many cases of pneumonia terminate naturally and spontaneously, so to speak, in health, quite independent [independently] of any active medical treatment. This is not a matter of inference merely, or probability, but of positive certainty, ascertained by ample and conclusive observation. I need here only refer to the cases reported by M. Grisolle. *How many* cases would so terminate, it is of course quite impossible to say. The proportion is probably large ; it is certainly very considerable. For instance, every thing that we know of the disease leads to the conclusion that nearly all cases of moderate or average severity, occurring from the eighth or tenth to the twenty-fifth or thirtieth year of life, in persons of sound constitutions, and up to the time of attack in good health, would, under the ordinary precautions of rest, regimen, and so on, terminate spontaneously in recovery. Such, at least, is the general tendency and the general result in these cases. This conclusion rests upon the fact, that many such cases have been known to recover without any active treatment ; and that under various, and in some degree, opposite modes of treatment, the mortality in this class of patients is almost always very small.

"In the second place, it is as certain that some cases of the disease will terminate fatally, notwithstanding any assistance which art may furnish, and in defiance of this assistance. Under all modes of treatment—positive or negative—heroic or expectant—that have ever been adopted, however skilfully and judiciously applied, numerous cases of the disease have always terminated, and still continue to terminate, in death. In the actual state of our knowledge, there are many cases over which art has little or no control; at least, not enough to prevent their fatal issue. And here it is important to observe, that everything in the natural history of pneumonia, and in the analogies derived from other diseases, leads to the conclusion that these cases are, as Sir Gilbert Blane said of the worst forms of yellow-fever, '*determinedly fatal*,'—terminating, not contingently and accidentally, or through want of knowledge and skill, but necessarily and inevitably, in death. There is good reason for believing, not that they are fatal through our ignorance—merely because we have not yet discovered the means of curing them—but that they are fatal in the nature of things, and by their own constitution. There is every reason for believing, not that we have failed to find the means of curing them, but that there are no such means."

The sum of the argument is, that in some cases the disease "terminates naturally and spontaneously, so to speak, in health, quite independently of any active treatment;" and that there are others which are "determinedly fatal"—"terminating, not contingently and accidentally, or through want of knowledge and skill, but necessarily and inevitably, in death;" and, "Finally," says our authour, "there is a third class of cases, occupying the ground between the two foregoing classes, and not tending necessarily in one direction or the other—not terminating naturally in recovery, nor inevitably in death. It may be impossible to predicate this of any given individual case of disease; indeed, the very constitution of this class of cases renders it so. Their termination is contingent; it depends upon circumstances; and we cannot know, absolutely, that any given case of this class, terminating in death, would, under any circumstances, have terminated otherwise." So it would appear, from the author's shewing, that the certainty of medicine consists in its uncertainty!

We have only room for one more extract. It is the concluding part of the author's peroration; and if every one who reads it, when he arrives at "*the everlasting stars*," does not throw up his cap, and shout long live the profession, and long live Dr. Bartlett, there is no use in the axle of an author's car waxing fervid and flashing light from its flying wheels!

"With these convictions of the powers and capabilities of our art, and of the general worthiness of its practitioners, we may rest assured, if we are only true to ourselves and to it, that the regard in which it has been held since the days of Hippocrates is in no danger of being permanently withdrawn. We must needs be visited occasionally by medical as by manifold other delusions; but it is a part of their nature always to pass rapidly away, and to be soon forgotten. They are like fluttering eddies that cross the main current of the Mississippi or the Amazon; to him who happens to be caught in the tiny whirlpools they may seem like the majestic tide of the great river itself, but they are soon inevitably lost and swallowed up in the rush of its resistless waters, to appear and be seen no more. No, there is no danger. The work of two thousand years is not to be demolished by the noisy clamor of a few penny trumpets. As certainly as there is truth in the foregoing inquiry, will the present feeling of distrust towards our science and our art pass away. The ancient confidence will be restored; the old love will come back again, truer and deeper for the transient and passing estrangement. The constellations themselves—Orion and the Pleiades—are sometimes apparently blotted out from the heavens by the gorgeous glare of rockets and other artificial fireworks, kindled with sulphurous and nitrous compounds; but, courage! my friends, and a little patience—the show will soon be over; the parti-coloured flame that would rival and eclipse the planets is even now dying away; all that will remain of the blazing illumination will be some noisome gases in the atmosphere, and a few burnt out sticks on the ground; but lo! still looking down upon us, with their dear old smile of affectionate recognition, from their blue depths in the firmament, undimmed in their brightness and unchangeable in their beauty, the everlasting stars."

